# Evaluation of a Worksite Cervical Screening Initiative to Increase Pap Smear Uptake in Malaysia: A Cluster Randomized Controlled Trial

**DOI:** 10.1155/2013/572126

**Published:** 2013-09-02

**Authors:** Fauziah Abdullah, Michael O'Rorke, Liam Murray, Tin Tin Su

**Affiliations:** ^1^Ministry of Health Malaysia, 62590 Putrajaya, Malaysia; ^2^Centre for Population Health (CePH), Department of Social and Preventive Medicine, Faculty of Medicine, University of Malaya, 50603 Kuala Lumpur, Malaysia; ^3^Centre for Public Health, School of Medicine, Dentistry and Biomedical Sciences, Queen's University of Belfast, Belfast BT12 6BA, Ireland

## Abstract

*Background*. Despite the significant burden of cervical cancer, Malaysia like many middle-income countries relies on opportunistic cervical screening as opposed to a more organized population-based program. The aim of this study was to ascertain the effectiveness of a worksite screening initiative upon Papanicolaou smear test (Pap test) uptake among educated working women in Malaysia. *Methods*. 403 female teachers who never or infrequently attended for a Pap test from 40 public secondary schools in Kuala Lumpur were recruited into a cluster randomized trial conducted between January and November 2010. The intervention group participated in a worksite cervical screening initiative whilst the control group received usual care from the existing cervical screening program. Multivariate logistic regression was performed to determine the impact of the intervention program on Pap smear uptake after 24 weeks of followup. *Results*. The proportion of women attending for a Pap test was significantly higher in the intervention than in the control group (18.1% versus 10.1%, *P* value < 0.05) with the worksite screening initiative doubling the Pap smear uptake, adjusted odds ratio 2.44 (95% CI: 1.29–4.62). *Conclusion*. Worksite health promotion interventions can effectively increase cervical smear uptake rates among eligible workers in middle-income countries. Policy makers and health care providers in these countries should include such interventions in strategies for reducing cervical cancer burden. This trial is registered with  IRCT201103186088N1.

## 1. Introduction

Despite the availability of effective screening tools, cervical cancer remains the second leading cancer among Malaysian women with 4,057 cases reported between 2003 and 2005 [1]. The age-standardized incidence rate for cervical cancer is 12.2 per 100,000 population, increasing with age after 30 years with rates highest amongst Chinese and Indian ethnicities [2]. Over 80% of cervical cancer patients present with advanced stage disease [3] and consequently have a poor survival rate [4, 5].

The Papanicolaou smear test (Pap test) has been recognized as a primary screening tool for the early detection of cervical cancer since the 1940s, implemented worldwide through either opportunistic or organized population-based approaches [6]. In opportunistic screening, the service is provided to women who request it or who attend a health facility for other services. In an organized program, eligible women are identified, tracked, and regularly invited for screening tests. 

Organized population-based programs are practiced in many developed countries such as Australia, New Zealand, Korea, Singapore, the Nordic countries, and the United Kingdom as well as several middle-income countries in Latin America and Eastern Europe [7–9]. Thus, these countries experience lower cervical cancer incidence and mortality as a consequence of enhanced screening coverage [8, 10]. Malaysia has employed an opportunistic cervical screening program since the 1960s; however, Pap smear coverage and the incidence of cervical cancer have not been satisfactorily improved [11, 12].

Developing countries such as Malaysia face many challenges in implementing nationwide call-recall cervical screening initiatives, largely due to limited resources and the absence of a Pap smear register to trace eligible women [13–15]. A possible solution may be to implement worksite cervical screening programs wherein eligible women can be easily tracked and invited to have a Pap test [16, 17]. However, there is insufficient scientific evidence of the feasibility and effectiveness of implementing such a screening practice in worksites in middle-income countries. 

A cluster randomized trial was undertaken to ascertain the effectiveness of a worksite screening initiative upon Pap test uptake among secondary school teachers of reproductive age in the state of Kuala Lumpur. 

## 2. Methods

### 2.1. Study Design and Participants

The study was a two-armed, parallel group, unblinded, cluster randomized controlled trial. There are 84 national secondary schools in Kuala Lumpur; these can be divided into four zones with an average of 20 schools within each zone. Ten schools were selected at random from each of these zones and randomized (1 : 1) to the intervention or nonintervention arms of the trial. Female secondary teachers who were either naïve to Pap smear or had their last test more than three years previously were invited to participate in the study (*n* = 403). Reasons for selecting this population as a setting stemmed from earlier research which had noted a low prevalence of Pap smear uptake in unemployed women, particularly tertiary educated women in the state of Kuala Lumpur [11, 18]. 

Ethics approval was obtained from the University Malaya Medical Centre (UMMC) Ethics Committee, Ministry of Education of Malaysia and Ministry of Health of Malaysia. This trial was registered at the Iranian Registry of Clinical Trials (IRCT), registration number: 201103186088N1.

### 2.2. Sample Size

Two previous studies conducted in Sweden [19] and Mexico [20] have reported the proportion of women attending cervical screening following an invitation and/or reminder to be in the order of 31%. Sample size was calculated using OpenEpi v.2.3.1 [21]; based on a 1 : 1 ratio of case to control participants, the study would need approximately 200 participants (inclusive of an additional 20% to account for non-respondents and design effect of two [22]) in the intervention and comparator arm to have over 80% power at the 95% confidence level (alpha 0.05, two sided) to detect an effect size of 31%. 

### 2.3. Data Collection

Data were collected in 10 months, from January to November 2010. A pretested self-administered baseline questionnaire was sent to 403 eligible women who fulfilled the inclusion criteria and consented to participate the study. The questionnaire was bilingual, containing both Malay (the national language) and English language and collected information on five key domains: (i) demographic and socioeconomic factors; (ii) reproductive history; (iii) healthy lifestyle and risk behaviour; (iv) attitudes and beliefs of cervical cancer screening practice; (v) stages of cervical screening behaviour change. A postintervention questionnaire was administered at 24 weeks to the intervention and comparator groups to identify women who had underwent Pap smear (primary outcome measure). 

## 3. Randomization

Using a computer generated simple randomization method in SPSSv15 (SPSS, Inc., Chicago, IL, USA), each of the 40 public secondary schools which had agreed to participate were randomized into either the intervention (*n* = 20) or control (*n* = 20) groups whereby all teachers from the same school (cluster) were assigned to the same group. Randomization was revealed after recruitment of the final school to ensure concealment of allocation. 

### 3.1. Intervention

The worksite screening intervention consisted of an invitation letter and information pamphlet on cervical cancer and purpose of Pap smear testing. This was followed up after four weeks by a short telephone reminder (performed once for each participant) which served to reiterate the importance of Pap smear testing on earlier cervical cancer detection. The invitation letters were personally distributed to each participant via the principal of each respective school. 

### 3.2. Statistical Analysis

Baseline characteristics were compared using a *t*-test for continuous variables and a chi-squared test for categorical data. Multivariate logistic regression was applied to calculate odds ratios (ORs) and 95% confidence intervals (CIs) for the impact of the worksite cervical screening program on the uptake of Pap smear testing. Variables from univariate analysis (irrespective of the intervention status) which had a *P* value ≤ 0.25 were selected for the multivariate model. Collinearity among the variables in the multivariate model was checked and determined absent. The value from the Hosmer-Lemeshow goodness-of-fit test of the multivariate logistic regression model was 3.74 (df = 8, *P* value = 0.880) indicating a well-calibrated model. SPSS v.15.0 (SPSS, Inc., Chicago, IL, USA) was used for all analyses. 

## 4. Results

**Figure 1:**
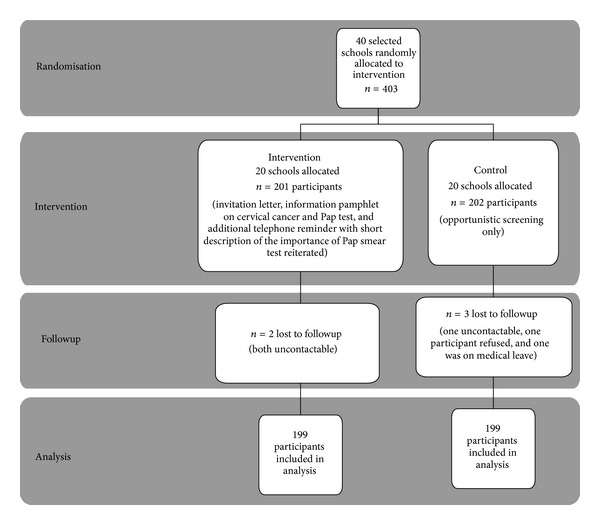
Flow of participants in the intervention and control arm.

Twenty secondary schools were allocated to receive the worksite cervical screening intervention (*n* = 201 eligible participants) and 20 schools (*n* = 202 eligible participants) were assigned to the standard (opportunistic) cervical screening arm. Both groups were followed up after a period of 24 weeks, at which point subsequent uptake of Pap examination was examined. Two participants from the intervention arm (both of whom were un-contactable) and three from the control arm (one uncontactable, one refused participation and one could not participate due to medical leave) were lost to followup, leaving 398 women for analysis (*n* = 199 participants in each arm). The flow of participants from both groups is outlined in Figure 1.

Table 1 compares the baseline characteristics of the intervention and control group. The majority of participants were Malay, Muslim, married (once only), had a graduate degree, multiparous, first pregnant at an older age, not on contraception, and had no genital symptoms. Most of the participants were overweight or obese, were never smokers, and did not meet physical activity recommendations, did not practice monthly breast self-examination, and the majority were too young (<50 years) to seek regular mammography and relatively few had concomitant chronic diseases. The majority of participants had never had sexual health education. Interestingly, the proportion of participants reporting ever having a Pap smear test was just 34.3% in the intervention arm and 41.6% in the control group. Despite this, 43.8% and 55.4% of participants stated that they were receptive to having a Pap smear test within the next six months in the intervention and control group, respectively. 

### 4.1. Effectiveness of the Worksite Cervical Cancer Screening Initiative

After 24 weeks of followup, a higher proportion of participants in the intervention arm (18.1%) were found to have attended for Pap smear testing than the control group (10.1%), representing an absolute increase in Pap smear uptake of 8% between the intervention and control group. Upon multivariate logistic regression analysis, Pap smear uptake was twice as high among those women in the intervention arm compared to the control group OR 2.44 (95% CI 1.29, 4.62). It is estimated that 12 women would need to receive the worksite cervical screening intervention in order for one woman to avail of cervical cancer screening (Table 2).

## 5. Discussion

This cluster randomized trial of a worksite cervical screening initiative, the first in Malaysia, has shown that by including a simple modification (invitation and reminder component) to the existing opportunistic screening program in Kuala Lumpur, a significantly greater proportion of women attended for subsequent Pap smear testing, a result which is consistent with previous study findings worldwide [17, 19, 23–26]. 

In the present study, an absolute increase in Pap smear uptake of 8% was observed between the intervention and control arm with inclusion of a written invitation and telephone reminder to attend screening. Elsewhere in the literature, whilst inclusion of an invitation or reminder system has been shown to significantly augment cervical screening attendance, the magnitude of effects across the randomized trials has varied. For instance, a population-based study in Sweden [19] observed Pap smear increases of just 1.3% with a written invitation (and information pamphlet); notably, however, Pap smear uptake increased to 9.2% and 31.4% with a written reminder and telephone reminder, respectively. Conversely, a telephone support intervention to improve cervical screening uptake in New York (USA) [27], reported a much smaller increase in Pap smear uptake of 0.07%. A study in New South Wales reported a 2% increase in Pap smear uptake with the addition of a reminder letter [28], whilst a similar intervention study in Belgium observed a 6.4% increase [26]. In Canada, introduction of a call-recall system increased cervical screening compliance by 4.4% [29]. The variation in Pap smear uptakes between the previous studies and the present analysis may be explained by differences in recruitment strategy methods used in inviting women for the Pap test, background characteristics of the study populations, and the effect of peers and frequent contact among participants in a natural setting like schools which may have encouraged participants to advocate each other for screening practice. 

Importantly, cervical cancer is a largely preventable disease. The Human papillomavirus (HPV) has been shown to account for more than 99% of all cervical cancers, the highest attributable fraction ever identified for a specific cause of cancer [30]. Importantly, in countries with an organized Pap smear screening program the incidence of invasive cervical cancer has been shown to be reduced by as much as 80% [9]. The current opportunistic screening program was employed in Malaysia in 1969, but like many middle income countries the implementation of this initiative has highlighted many caveats [3, 14, 31–33], not least of which has been an inadequate population coverage [3, 14, 34, 35]. In 1995, the importance of Pap smear screening was emphasized through the launch of a “healthy lifestyle campaign against cancer” by the Ministry of Health which made Pap smear testing freely available for all women aged 20–65 years of age once every three years. Unfortunately, uptake of Pap smear testing has remained low, estimated to be around 47.3% at the Third National Health and Morbidity Survey in 2006 [13]; however, the actual Pap smear coverage in Malaysia remains unknown due to a lack of a Pap smear registry. 

Despite the example set by other middle-income developing countries in Latin America and Eastern Europe that have implemented or piloted an organized (population-based) call-recall cervical cancer initiative and which have succeeded in reducing cervical cancer incidence and deaths [36–38], policy makers in developing countries share the concern that their available resources may be inadequate for the implementation of such an approach. Therefore, until the feasibility and cost effectiveness of an organized call-recall screening program to reduce the incidence and mortality from invasive cervical cancer are realized [14], it may be prudent to consider alternative effective cervical screening initiatives. The findings of this study and a similar Swedish study [19] support the addition of an invitational component and systematic followup (personal telephone reminder) to the present opportunistic cervical screening program as key factors in encouraging women to practice Pap smear more frequently. Worksite cervical screening initiatives may therefore offer a promising alternative to enhance Pap smear coverage. In fact in 2005, 60% of Malaysian women were participating in the labour force [39] and the percentage of working women continues to increase. What is more is that some 80%–85% of these women's husbands participate in the labour workforce also, providing an opportunity for these men to extend a Pap smear invitation to women aged 20–65 in their household. 

To the authors knowledge, this is the first trial of a worksite cervical cancer screening initiative in Malaysia. The principle strength of this study is its randomized study design and ascertainment of a wide range of baseline sociodemographic, reproductive health and lifestyle information (which were demonstrated to be similar between the groups) combined with a very low loss to followup. Moreover the results of this study may be generalized to the urban setting of other middle-income countries in the Asian region. It is important to note that the findings herein pertain to well-educated professionals (female teachers) in secondary public schools of Kuala Lumpur, one of sixteen city states in Malaysia. Importantly, approximately half of cervical cancers in Malaysia occur in women who have attained only up to a primary level of education [13]; therefore, the women in this study may represent a lower risk group for cervical cancer. Moreover, the majority of women in this study were Malay (>80%) and married (98.5%), limiting the extrapolation of these findings to other ethnicities (Chinese and Indian women) and unmarried women. Interestingly, Pap smear uptake rate in this study, whilst significantly different in the intervention and control arm, was relatively low overall (14.1%), indicating that even among well-educated and informed women Pap smear screening is not often put into practice. A recent qualitative study among 20 Malaysian women aged 21–56 years has shown that women's knowledge, attitudes, and beliefs influenced their cervical screening behaviour [33]. The authors found that women were inadequately informed of the indications and benefits of cervical screening with many believing that the Pap smear detects existing cervical cancer or that they perceived themselves to be at a low risk for developing the cancer so did not attend for screening. It follows therefore that a concerted effort is needed to challenge these misconceptions and improve cervical cancer public knowledge and awareness. Other commonly cited barriers to cervical screening include psychosocial and cultural contexts, fear and limited family support [40]. 

In conclusion, as in many other countries, an organized call-recall cervical screening program in Malaysia has the potential to substantially lower the burden of cervical cancer incidence and mortality. However, until such a program is realized, worksite cervical screening initiatives (with both an invitation and follow-up component) may offer an alternate means of improving Pap smear uptake within the current opportunistic cervical screening practice. This alternate strategy based on worksite health promotion should be appreciated by policy makers and health care providers in middle-income countries as a promising intervention for reducing the long standing burden of cervical cancer in Malaysia. 

## Figures and Tables

**Table 1:** Baseline characteristics of women in the intervention and control group.

Variables	Intervention group (*n* = 201), %	Control group (*n* = 202), %	*P* value
Demographics			

Mean age (years) (±SD)	36.1 ± 8.0	36.5 ± 7.3	0.455^‡^
Ethnicity: Malay	85.1	83.2	0.376^‡^
Religion: Muslim	85.1	84.7	0.572^‡^
Marital status: married	98.5	98.5	1.000^‡‡^

Socioeconomic status			

Educational level			0.939^‡^
Diploma	2.5	2.5	
Graduate degree	90.0	89.1	
Master degree	7.5	8.4	
Mean household monthly income (MYR) (±SD)	7967.0 ± 8068.4	6777.8 ± 3469.1	0.486^*∂*^

Reproductive history			

Number of pregnancies (including abortion)			0.539^‡^
1–4	66.2	71.3	
≥5	21.5	18.8	
Never pregnant	11.9	9.9	
Mean number of pregnancies (±SD)	2.9 ± 2.0	2.9 ± 1.9	0.784^*∂*^
Practicing contraceptive currently: yes	30.3	32.7	0.615^‡^
Having any genital symptoms currently: no	82.1	82.7	0.878^‡^
Ever had Pap test (≥3 years ago)	34.3	41.6	0.133^‡^
Stages of cervical screening behavior change			0.051^‡^
Precontemplation	35.8	26.2	
Contemplation	43.8	55.4	
Preparation	20.4	18.3	

Physical health			

Body mass index (kg/m^2^)			0.944^‡^
Normal (18.5–22.9)	31.3	32.2	
Underweight (<18.5)	5.0	5.4	
Overweight and obese (≥23)	63.7	61.9	
Ever smoke: no	100.0	99.5	1.000^‡‡^
Practice physical activity as recommended: yes	10.9	9.4	0.609^‡^
Perform breast self-examination monthly: yes	45.8	48.0	0.651^‡^
Perform mammogram as recommended			0.202^‡^
Applicable	5.0	2.5	
Not applicable	67.2	63.4	
No	27.9	34.2	
Had medical checkup in last 3 years: yes	49.8	53.0	0.518^‡^
Having any chronic diseases: yes	19.4	22.8	0.407^‡^

Social health			

Number of marriages: once	99.0	98.5	1.000^‡‡^
Mean age of first marriage (years) (±SD)	25.5 ± 2.7	25.9 ± 2.9	0.082^*∂*^
Mean age of first sexual intercourse (years) (±SD)	25.6 ± 2.5	26.0 ± 2.9	0.123^*∂*^
Age of first pregnancy (years)			0.091^‡^
Never pregnant	11.9	9.9	
≤25	34.3	25.7	
>25	53.7	64.4	
Mean age of first pregnancy (years) (±SD)	23.2 ± 8.9	24.4 ± 8.6	0.063^*∂*^
Ever had a sexual health education: yes	34.8	35.6	0.864^‡^
Practicing conservative methods to prevent cervical cancer:			
Safe sex	94.5	96.0	0.474^‡^
Self-circumcised	83.6	82.2	0.708^‡^
Husband/partner circumcised	86.1	86.6	0.869^‡^
Prohibit extramarital sex	95.5	96.0	0.796^‡^
Having health insurance: yes	66.2	70.3	0.413^‡^

Notes: ±SD standard deviation, ^‡^chi square test, ^‡‡^Fisher's exact test, ^*∂*^ *t*-test, MYR denotes Malaysian ringgit (where 1 MYR equals 3 USD).

**Table 2:** Pap smear uptake within 24 weeks of followup in the intervention and control group.

	Participated in Pap smear screening test, *n* (%)	Univariate modeling		Multivariate modeling^a^	
		OR	95% CI	OR	95% CI
Intervention group (*n* = 199)	36 (18.1)	1.98	1.1–3.5*	2.44	1.29–4.62
Control group (*n* = 199)	20 (10.1)	1.0	(ref.)	1.0	(ref.)

Notes: ^a^Multivariate model was adjusted for the demographic characteristics, socioeconomic status, reproductive history, and lifestyle factors shown in Table 1, where the *P* value in univariate analysis was <0.25.

*P value < 0.05.

OR denotes odds ratio.

CI denotes confidence interval.

## Abbreviations

CI: Confidence interval
df: Degree of freedom
IRCT: Iranian Registry of Clinical Trials
OR: Odds ratio
UMMC: University Malaya of Medical Centre.
